# Vectorborne Transmission of *Leishmania infantum* from Hounds, United States

**DOI:** 10.3201/eid2112.141167

**Published:** 2015-12

**Authors:** Robert G. Schaut, Maricela Robles-Murguia, Rachel Juelsgaard, Kevin J. Esch, Lyric C. Bartholomay, Marcelo Ramalho-Ortigao, Christine A. Petersen

**Affiliations:** University of Iowa, Iowa City, Iowa, USA (R.G. Schaut, C.A. Petersen);; Kansas State University, Manhattan, Kansas, USA (M. Robles-Murguia, M. Ramalho-Ortigao);; Iowa State University, Ames, Iowa, USA (R. Juelsgaard, K.J. Esch);; University of Wisconsin, Madison, Wisconsin, USA (L.C. Bartholomay)

**Keywords:** zoonotic, emerging, vectorborne infections, canine, Leishmania, protozoa, parasites, United States, leishmaniasis, Leishmania infantum, hounds

## Abstract

Leishmaniasis is a zoonotic disease caused by predominantly vectorborne *Leishmania* spp. In the United States, canine visceral leishmaniasis is common among hounds, and *L. infantum* vertical transmission among hounds has been confirmed. We found that *L. infantum* from hounds remains infective in sandflies, underscoring the risk for human exposure by vectorborne transmission.

Leishmaniasis is endemic to 98 countries (*1*). Canids are the reservoir for zoonotic human visceral leishmaniasis (VL) (*2*), and canine VL was detected in the United States in 1980 (*3*). Subsequent investigation demonstrated that many US hounds were infected with *Leishmania infantum* (*4*). Evidence has demonstrated that *L. infantum* was spread by vertical transmission over many canine generations; no evidence of vector transmission has been reported (*5*,*6*). Vertical transmission may lead *L. infantum* to adapt to vectorless transmission and shed largely unrecognized factors needed for vector infection. Continuous axenic cell culture conditions without vector involvement have been shown to attenuate pathogen infectivity (*7*). Similarly, *L. infantum* circulating primarily via vertical transmission within US hunting hounds may lose its ability to infect and may be transmitted by traditional vectors.

In North America, 3 species of sandfly (*Lutzomyia anthophora, Lu. diabolica,* and *Lu. shannoni*) are known vectors of *Leishmania* spp. Reported cases of autochthonous cutaneous leishmaniasis in the United States include 9 cases in northeastern Texas (*8*), 2 in Oklahoma (*9*), and 1 in North Dakota (*10*). In the Americas, the principal sandfly vector is *Lu. longipalpis*, which can transmit *Leishmania* of multiple species. (*11*); its northernmost distribution is limited to Mexico. *Lu. shannoni* sandflies have been found in Kansas and Missouri (total range 21 states) (*12*). During 2010–2013, we assessed whether *L. infantum* circulating among hunting dogs in the United States can fully develop within sandflies and be transmitted to a susceptible vertebrate host. 

## The Study

**Figure 1:**
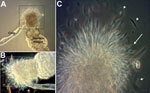
Sandflies infected with *Leishmania infantum* from US foxhounds, showing blocked stomodeal valve. Development of *L. infantum* (MCAN/US/2001/ FOXYMO1) in laboratory-reared *Lutzomyia longipalpis* sandflies led to stomodeal valve blockage 10–13 days after infection. A) Dissected gut of infected sandfly, showing stomodeal valve (cardia) obstructed by *Leishmania* parasites (dashed box). Foregut removed during dissection and parasites entangled by flagella are visible. Original magnification ×10. B) Parasites obstructing stomodeal valve and parasite-secreted plug (dashed box). Original magnification ×40. C) Parasite plug dissected from the stomodeal valve, showing metacyclic promastigote parasites attached to plug (arrow), as well as free-swimming parasites (arrowheads). Original magnification ×100 with oil.

A total of 300 laboratory-reared female *Lu. longipalpis* sandflies were allowed to feed on 2 hounds naturally infected with *L. infantum,* strain MCAN/US/2001/FOXYMO1 or a closely related strain. During 2007–2011, the hounds had been tested for infection with *Leishmania* spp. by ELISA, PCR, and Dual Path Platform Test (Chembio Diagnostic Systems, Inc. Medford, NY, USA (Table 1). *L. infantum* development in these sandflies was assessed by dissecting flies starting at 72 hours after feeding and every other day thereafter. Migration and attachment of parasites to the stomodeal valve of the sandfly and formation of a gel-like plug were evident at 10 days after feeding (Figure 1), indicating successful parasite development.

**Table 1:** *Leishmania infantum* status of US foxhounds on which infected sandflies fed*

Sex	Age, y	Type of test, date of testing					
		PCR, 2007	PCR, 2008	Serology/PCR, 2009	Serology/PCR, 2010	Serology/PCR, 2011	DPP, 2011
M	7	Borderline	–	–/–	32–	>512/+	+
F	6	–	+	64/–	>512/+	>512/+	+

*Serologic results determined by immunofluorescence antibody testing: <64 indicates negative (–). PCR results: – indicates no amplification; borderline indicates amplification on 1 of 3 tests; positive (+) indicates amplification on 2 of 3 or 3 of 3 tests. DPP indicates K39/22 Dual Path Platform Test (Chembio Diagnostic Systems, Inc. Medford, NY, USA), to detect antibodies against *Leishmania* spp.

Next, to determine sandfly capacity to transmit the US strain of *L. infantum* to a susceptible vertebrate host, we allowed *L. infantum*–naive and *L. infantum*–infected sandflies to feed on 7 *L. infantum*–naive hamsters for 13 days. For confirmation of *L. infantum* infection, we dissected the alimentary tract of sandflies that fed on the hamsters (Table 2). A total of 30 sandflies were used for feeding on hamsters; 11 flies fed and were subjected to *Leishmania* detection by PCR, which confirmed *Leishmania* positivity for 5 sandflies. Hamster blood samples were collected 2 weeks after infection and monthly for 5 months. *L. infantum* DNA was detected in hamster blood by quantitative PCR (qPCR) and was present in hamster nos. 1 (at 2 mo), 2 (at 3 mo), 5 (at 4 mo), and 6 (at 4 mo) with cycle thresholds of 43.88, 28.27, 34.38, and 45 respectively. Cycle thresholds <45 were considered positive for *L. infantum* (*5*). 

**Table 2:** Blood meal feeding and *Leishmania infantum* infection status of sandflies that fed on *L. infantum*–infected hamsters

Hamster no.	No. sandflies in which blood was visible/no. examined	PCR result for *L. infantum* from sandfly DNA extraction
1	1/5	–
2	2/5	+
3	1/5	–
4	0/5	Not applicable
5	3/5	2 + /1 –
6	2/5	1 +/ 1 –
7	2/5	–

On hamster no. 5, a cutaneous lesion consistent with *Leishmania* infection persisted for 1 month. Tissue from this lesion was harvested to assay for *Leishmania* infection. Increased numbers of macrophages and granulocytes were present in the dermal layer. Bacteria found in the tissue probably represented secondary infection, a common sequela of canine VL. Cellular infiltrate was observed, indicative of inflammation and infection (Technical Appendix). No *L. infantum* parasites were observed on slides stained with hematoxylin and eosin, periodic acid–Schiff, or Giemsa, and lesion tissue was negative for *L. infantum* by qPCR (data not shown).

**Figure 2:**
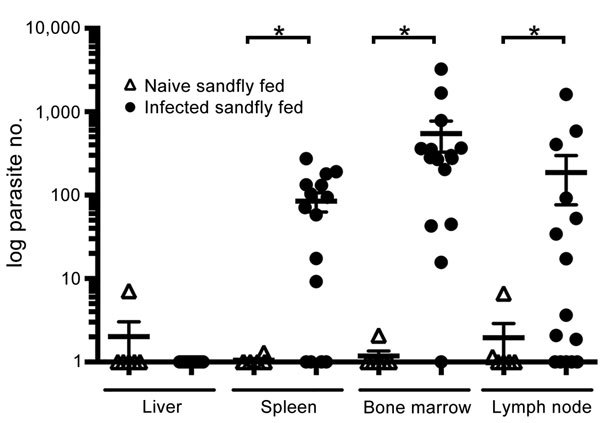
Visceralization of *Leishmania infantum* from US foxhounds, transmitted by sandflies into hamsters. *Leishmania* spp.–specific quantitative PCR was performed, and parasite load was calculated from a standard curve. Horizontal bars indicate mean values for 3 experiments run in duplicate. Statistical significance was determined by 1-way analysis of variance with Bonferroni posttest between 6 naive and 15 infected groups, by tissue type. Error bars indicate ± SEM. *p<0.05.

qPCR was performed to quantify parasite load within common *Leishmania*-infected organs from all hamsters. The mean quantities of *Leishmania* DNA amplified from spleen, bone marrow, and lymph node from hamsters on which *Leishmania*-infected sandflies had fed were 12-, 22-, and 11-fold greater than that from hamsters on which *Leishmania*-naive sandflies had fed (Figure 2). According to extrapolation from a PCR standard curve similar to one previously used (*5*), the highest parasite load was in bone marrow, which contained an average of 1,238 (±282) parasites/mg tissue. 

## Conclusions

In the United States, parasites from VL-symptomatic, naturally infected hunting hounds remain highly infectious to *Leishmania*-competent *Lu. longipalpis* sandflies. Parasites that had fed on *L. infantum*–infected hounds were able to develop fully within sandflies and to be subsequently transmitted to and disseminated within hamsters.

The capacity of *Lu. shannoni* and *Lu. longipalpis* sandflies to acquire *L. infantum* from naturally infected dogs has been compared in leishmaniasis-endemic Brazil (*13*). Although lower infection rates were observed in *Lu. shannoni* (9%) than in *Lu. longipalpis* (36%) sandflies, the intensity of infection (200–500 promastigotes/fly) was higher in *Lu. shannoni* sandflies*.* This finding demonstrates a potentially lower threshold for acquiring infection from *Lu. shannoni* sandflies because the infectious dose per sandfly was greater. *Lu. shannoni* sandflies are commonly found within the United States and also in areas where *L. infantum*–infected hounds were reported (*4*). These data demonstrate the risk for vectorborne transmission of zoonotic VL from these dogs in the United States.

Despite our use of an *L. infantum* strain that is primarily, if not solely, transmitted via vertical transmission between dogs in the United States, we were able to measure substantial parasite loads in sandflies that fed on these dogs and in the bone marrow, spleen, and peripheral lymph nodes of hamsters on which infected sandflies had fed (Figures 1, 2). Parasite DNA was not amplified in the liver, possibly because of lower parasite loads in the liver during later infection, as demonstrated in experimental VL infections of mice (*14*). Therefore, the US strain of *L. infantum* that is circulating in North American hunting hounds has not lost virulence factors that facilitate adherence to sandfly gut and facilitate transmission, and subsequent dissemination, in a secondary host.

This study focused on the possibility that domestic hounds serve as reservoir hosts for *L. infantum* within the United States; however, other potential *L. infantum* reservoirs include coyotes, foxes, and opossums. When leishmaniasis was found to be reemerging among hounds in the United States in 2000, a total of 291 wild canids were trapped and tested (*15*). No serologic evidence of infection was found, but these studies were limited to the southeastern United States; further study is needed to rule out the possibility that enzootic cycles of transmission do not exist within wild canids. The range covered by *Lu. shannoni* sandflies overlaps that of reservoir species including coyotes, foxes, and hunting hounds. Occurrence of *Leishmania* vectors in areas of naturally infected hounds indicates a coalescence of components for establishment of a sylvatic and/or domestic cycle of *L. infantum*. Diagnostic testing and preventive measures should be considered for dog breeds known to harbor *L. infantum*.

In the United States, *L. infantum* is circulating among dogs. Despite the fact that vertical transmission maintains VL within the hound population (*5*), *L. infantum* was able to fully develop in sandflies and be further transmitted to a susceptible vertebrate host. Symptomatic hounds were highly infectious to sandflies. *L. infantum* strain MCAN/US/2001/FOXYMO1, similar to the common European zymodeme MON-1, circulating dog-to-dog in North America maintained all necessary requirements for complete development within sandflies. Overlap of sandfly infections (e.g., *Lu. shannoni*, and *L. infantumi*) in hounds may put companion dogs and humans at risk and could pose an emerging risk for *L. infantum*–triggered clinical disease in at-risk populations in North America.

**Technical Appendix.** Photograph and histologic images of lesion on hamster on which *Leishmania infantum*–infected sandflies fed.
