# Supplementary material for: Vectorborne Transmission of Leishmania infantum from Hounds, United States
Source: Emerg Infect Dis. 2015 Dec;21(12):2209–12. doi: 10.3201/eid2112.141167 (PMC4672406; doi:10.3201/eid2112.141167)
Supplement: Supplementary file 1 — Technical Appendix. Photograph and histologic images of lesion on hamster on which Leishmania infantum–infected sandflies fed. [file 14-1167-Techapp-s1.pdf]

Article DOI: http://dx.doi.org/10.3201/eid2112.141167

Vectorborne Transmission of *Leishmania infantum* from Hounds, United States

Technical Appendix

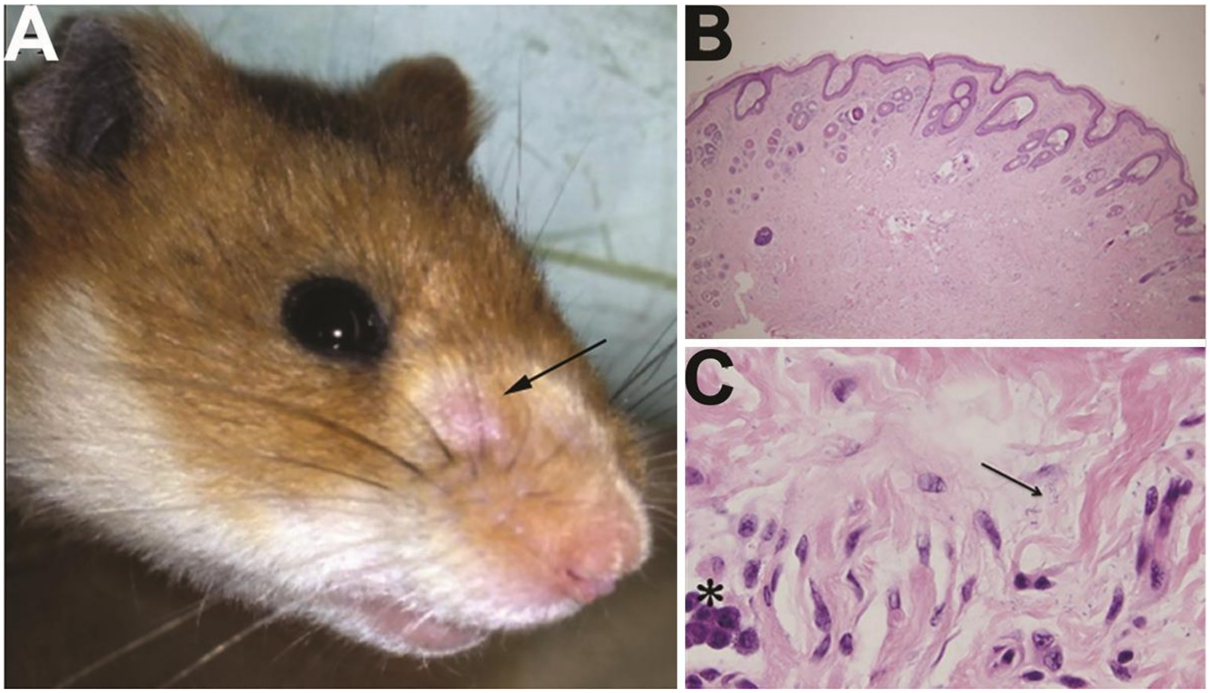

Technical Appendix Figure. A) Photograph of rostral portion of hamster no. 5, on which 2 sandflies infected with US foxhound strain of *Leishmania infantum* had fed; image taken 4 months after sandfly feeding. Arrow indicates skin lesion consistent with leishmaniasis. B) Histologic image of hematoxylin and eosin–stained cutaneous lesion demonstrating inflammation of the dermis with macrophage and mast cell infiltration. Original magnification ×100 with oil. C) Histologic image of hematoxylin and eosin–stained cutaneous lesion. Rod-shaped bacteria (arrow) within the deep dermis and mast cell (asterisk) infiltrates. Original magnification ×100 with oil.
